# Sarcopenia and its determinants among Iranian elderly (SARIR): study protocol

**DOI:** 10.1186/2251-6581-11-23

**Published:** 2012-11-21

**Authors:** Rezvan Hashemi, Ramin Heshmat, Ahmadreza Dorosty Motlagh, Moloud Payab, Ahmad Esmaillzadeh, Fereshteh Baigy, Parvin Pasalar, Fereydoun Siassi

**Affiliations:** 1 Department of nutrition and biochemistry, Faculty of public health, Tehran University of Medical Sciences, Tehran, Iran; 2 Endocrinology and metabolic research center, and chronic disease research center, Tehran University of Medical Science, Tehran, Iran; 3 Department of community nutrition, school of nutrition and food science, food security research center, Isfahan University of Medical Sciences, Isfahan, Iran; 4 Department of Biochemistry, Tehran University of Medical Sciences, Tehran, Iran

**Keywords:** Sarcopenia, Dietary pattern, Metabolic syndrome, Inflammatory marker

## Abstract

**Background:**

The elderly populations increase in world because of improved health status in communities, so health and independency of seniors has become and will be one of the main priorities of public health systems.

Ageing have been associated with changes in body composition, including loss of muscle mass, loss of bone mass and increase fat mass. Involuntary age related loss of muscle mass, sarcopenia,has been linked to functional impairment and physical disability. Several definitions for sarcopenia have been presented based on the method of measuring body composition, but an internationally accepted definition doesn’t presently exist yet.

In 2010, the European working group on sarcopenia developed a new definition for sarcopenia according to measure muscle mass and muscle function. Several studies have been done about sarcopenia in world, but to our knowledge this study is the first in Iran which is one of the largest countries of the Middle East that faces a fast growing elderly population. The aim of this study is to evaluate sarcopenia and related risk factors in Iran according new definition of sarcopenia.

**Methods:**

This study will be conducted in two phase among elderly men and women over 55 years in the 6^th^ district of TehranThe first phase will be a population-based cross-sectional study to determine the frequency of sarcopenia in the study population, and to conduct case finding for the second phase. The second phase will be a case–control study to comparison the metabolic and inflammatory factors in sarcopenic and non sarcopenic groups.

The association between sarcopenia and major dietary pattern will be evaluated using factor analysis.

**Conclusion:**

This study is the first study that evaluates sarcopenia and its risk factor in Iranian elderlies.

We discuss details of how we collect the data and appropriate instruments to measure muscle mass, muscle power and muscle strength, and suitable cut- off to define sarcopenia in Iranian elderlies. We believe the result of our study can be useful to health policy makers prepare the necessary infrastructure for elderly health improvements and increase the quality of life in geriatric.

## Background

The world’s population is aging. It is estimated that by the year 2050, the elderly population will triple from 600 million as it was in the year 2000 to more than two billion [1]. As a result, improving the health and independency of seniors has become and will be one of the main priorities of public health systems. World Health Organization (WHO) has named 2012 the Year of Health and Aging.

Aging is associated with changes in all organs including body composition, skeletal muscle, and bone mass [2-4]. Muscle mass decreases approximately 3–8% per decade after the age of 30 and this rate of decline is even higher after the age of 60 [5]. This involuntary age related loss of muscle mass was named for the first time in 1989 by Irwin Rosenberg as Sarcopeny. The term Sarcopenia consists of the Greek words *sarks* (flesh) and *penia (*loss) [6] and is equivalent to a process that occurs during osteoporosis [7].

Sarcopenia imposes significant costs on the health care system each year. It is the underlying cause of frailty [8], the debilitating syndrome in aging, and the sixth cause of death in people over 65 years [9]. Furthermore, Sarcopenia is a major risk factor of falling and disability in the elderly [10]. Functional impairment and physical disability in Sarcopenic people are 2 to 3 times more likely. In the United States, costs related to complications of Sarcopenia was estimated to be more than 18.5 billion dollars in 2000 [11].

Several studies have discussed the mechanisms involved in the development of Sarcopenia including alterations in sex hormones, a decrease in protein synthesis, neuromuscular integrity changes, an increase in muscle fat content, resistance to insulin, inappropriate physical activity and inadequate nutrition [12]. Additionally, other studies have emphasized the genetic role [13], inflammation, and oxidative stress [14] in development of Sarcopenia.

Since 1989, several definitions for Sarcopenia have been presented based on the method of measuring body composition. These methods include using bioempedance analysis, dual x-ray absorptiometry, computed tomography, magnetic resonance and measurement of total or partial body potassium [15]. However, an internationally accepted definition of Sarcopenia doesn’t presently exist yet [16]. In 2010, the European Working Group on Sarcopenia (EWGSOP) developed a new definition for Sarcopenia. EWGSOP recommends using the presence of both low muscle mass and low muscle function (strength or performance) for diagnosis of Sarcopenia in clinical and research tests [15]. EWGSOP suggests three stages for Sarcopenia: The Pre-Sarcopenia stage is characterized by low muscle mass without change in muscle strength or performance. The second stage, Sarcopenia, is defined by low muscle mass plus low muscle strength or low physical performance. The third stage, known as severe Sarcopenia, is associated with the decrease of all of three components, muscle mass, strength, and performance.

Researchers have studied the prevalence of sarcopenia in various countries around the world including developing countries [17-20]. We try to use (EWGSOP) definition to study sacopenia in a country which has not been studied before. We focus on Iran which is one the largest countries of the Middle East and is dealing a fast growing elderly population. Our main objective is to measure the prevalence of sarcopenia among Iranian elderlies using the EWGSOP definition. In addition, we try to evaluate the risk factors associated with sarcopenia in our targeted population. Ultimately we aim to study the role of diet, metabolic syndrome and inflammatory markers on sarcopenia. This will help us recognize the modifiable risk factors in dietary pattern of elderly Iranians which in turn can be used in dietary recommendations for sarcopenia prevention.

## Method

### Design and setting

This study will be conducted in collaboration with the Nutrition School, Endocrinology and Metabolism Research Institute of Tehran University of Medical Sciences.

The project is consisted of two phases.

### First phase

The first phase is a population-based cross-sectional study, which will be conducted among elderly men and women in the 6^th^ district of Tehran ^a^[21] in order to determine the frequency of Sarcopenia in the study population and to conduct case findings for the second phase.

### Second phase

The second phase will be a case–control study. The case group includes participants with Sarcopenic criteria in accordance with EWGSOP definition (prevalent cases). Participants in the first phase who lacked the necessary criteria for Sarcopenia will make up the control group.

### Participants

Using cluster random sampling, 30 clusters in the 6^th^ region of the Tehran Municipality will be determined in collaboration with the Iran post office. The head of the clusters will be selected based on a ten-digit postal code and subjects will be asked for home interviews. Sampling will be continued in each cluster in clockwise order until the desired sample size is reached.

In each cluster, two individuals (one male, one female) will be invited from each of the following age groups: 55–59, 60–64, 65–69, 70–74, and over 75 (a total of 10 persons in each cluster). During the home interviews, these individuals will be briefed about the project and its objectives and for those who agree to participate, clinic appointments will be set. Participants will be asked to comply with the following requirements prior to their appointments: a) Participants should not be pregnant. b) Participants will be required to fast for 10 hours prior to testing. c) Participants should not take calcium supplements two days prior to testing. d) Metal objects (e.g. piercing, earrings, jewelry)are not allowed. e) Participant should bring his/her medications.

### Inclusion criteria

1.Participants should be at least 55 years old.

2.Participants will be required to have the ability to move without crutches, walker or other assistive devices.

3.There should be an absence of artificial limbs or limb prosthesis.

4.There should be an absence of active cancer, according to individual self report.

5.Three should be an absence of Congestive Heart Failure (CHF), Chronic Obstructive Pulmonary Disorder (COPD), Chronic Renal Failure (CRF), cirrhosis and liver failure (all based on the individual self report).

### First phase sample size

The population size will be 300 persons (power 80%, design effect 1.2, α =%5).

### Second phase sample size

Our case group (prevalent cases) will consist of those participants who are considered Sarcopenic based on the EWGSOP definition in phase 1. Each participant in the case group will then be matched with two participants from the same age group who are not considered Sarcopenic using the same EWGSOP definition.

The minimum sample size for the case group will be 30 persons, according to the comparison of protein intake between Appendicular Lean Mass’s first and second quartiles [22]. The minimum sample size for the control group will be 60.

The study protocol will be reviewed by Tehran University of Medical Sciences ethics committee.

## Measurements

The following measures will be performed for each individual in the clinic after obtaining a written consent: a 10 cc fasting blood sample will be taken for assessing metabolic syndrome constituent. This consists of fasting blood sugar(FBS), triglyceride(TG), and high density lipoprotein (HDL) in first phase. Some portion of the serum sample (2 ml) will be kept at −80°C for further examinations in the second phase.

### Questionnaires

Three questionnaires including, a general questionnaire, a physical activity questionnaire, and a food frequency questionnaire (FFQ) will be completed for each participant by a trained dietitian.

Participants will be asked about age, marital status, education, past medical history, smoking, and alcohol use in general questionnaires. The past medical history includes a history of diseases such as stroke, myocardial infarction, asthma, diabetes, and arthritis, as well as a history of drug consumption such as oral sexual hormones, statins, angiotensin convertin enzyme inhibitors, and insulin. In order to adjust their effect on inflammation and the metabolic syndrome, participants will also be asked about the consumption of aspirin, oral glucose-lowering drugs, blood pressure-lowering drugs, corticosteroid and lipid lowering drugs.

The physical activity level will be evaluated by a short form physical activity questionnaire (IPAQ) [23], which will be translated into Persian. Participants will be asked to report time spent on walking, moderate-intensity activity, and vigorous-intensity activity during the week prior to test. The physical activity data will be converted to minute per week and expressed as a metabolic equivalent (MET-min/week) according to IPAQ guidelines for data processing [24]. The participants with total physical activity lower than 600 MET-minute/week will be considered as low physical activity, the amount above 3000 MET-minute/week will be considered as high physical activity, and the amount between 600 to 3000 will be considered as moderate physical activity.

The participants’ dietary intake will be assessed by using 117 items in the semi-quantitative FFQ. The FFQ consists of a list of food items with standard serving sizes commonly used by Iranian consumers. Participants will be asked to report their consumption frequency of each food item during the year prior to the test according to daily, weekly and monthly intervals. This FFQ has been validated for 40–60 year old female residents of Tehran by Esmaillzadeh et al. [25]. To examine the validity and reliability of FFQ in the older population, we have designed a pilot study.

### Anthropometric and blood pressure measurement

Height will be measured in meters using a wall tape in standing position without shoes. Participants’ weight (in kg) will be assessed using a digital scale, while they are minimally clothed. Waist circumference will be measured in the middle of lower rib margin and iliac crest, standing and breathing normally. A general physician will measure participants’ blood pressure in the sitting position after 15 minutes rest using an analog sphygmomanometer. The first sound appearance will be considered as systolic blood pressure, and the disappearance of sound will be diastolic blood pressure.

### Body composition analysis

Muscle mass can be measured using a wide range of devices such as magnetic resonance imaging, computed tomography, dual x-ray absorptiometry, bioimpedance analysis, and total or partial body potassium in lean mass [15]. DXA is an attractive alternative method for research and clinical use to distinguish fat, bone and lean tissues [15]. This device can measure fat mass, muscle mass, and bone mass of head, trunk, and extremities separately with minimal radiation exposure. In this study we will use the DXA scanner (Discovery W S/N 84430) to determine body composition for each person. Participants will be ask to lie supine without movement during imaging. The time which is required for evaluation of each participant will be 15 minutes. According to DXA results, we will calculate the appendicular skeletal muscle mass for each participants as the sum of upper and lower limb muscle mass (in kg) [26]. Since a large proportion of total body muscle mass is found in appendicular skeletal muscle, AST can accurately represent the total muscle mass [27]. In order to eliminate the effect of height on total muscle mass, the relative skeletal muscle mass will be computed as the ratio of ASM to squared height (ASM/height^2^) [6]. Based on the findings of other studies in the literature, the relative skeletal muscle mass less than 7.26 kg/m^2^ for men and 5.5 kg/m^2^ for women will be considered abnormal [28].

### Muscle strength measurement

There are several techniques for estimation of the muscle strength, such as handgrip strength, knee flexion/extension, and peak expiratory flow. Isometric grip strength is a good simple measure of muscle strength, which is known to be strongly correlated with leg strength [15]. Isometric grip strength can be measured by two well known devices, the Jamar dynamometer and the Martin vigorimeter [29]. Unfortunately, because of the lack of access to both of these devices, we will have to use a squeeze bulb dynamometer (c7489-02 Rolyan), which is a pneumatic instrument like a modified sphygmomanometer and is calibrated in pounds per square inch (psi). The squeeze bulb dynamometer measures isotonic muscular action instead of isometric strength, which is measured by the widely used Jamar dynamometer or Martin vigorimeter. However, studies have shown a strong correlation between the two measurements [30].

In accordance with the recommendations of American Society of Hand Therapists, we will measure grip strength with maximum voluntary contractions for each participant. We will then repeat the measurement three times for each hand with a 30-second rest time in between each trial. The average of maximum values for the left and right hands will be considered as the measure of the participant’s muscle strength. The cutoff value for muscle strength will be obtained from previous studies [31].

### Muscle performance

Several tests have been suggested in the literature for evaluating muscle performance including, Short Physical Performance Battery (SPPB), usual gait speed, 6-min walk test and the stair climb power test. We will evaluate participants’ muscle power by performing a 4-m course gait speed test which is a part of SPPB, but it can also be used as a single parameter for clinical practice and research [15]. Each participant will be asked to walk at his/her usual pace to the other end of the 4-meter course. The time will be recorded by chronometer in seconds. Participants with gait speeds lower than 0.8 m/s will be considered to be at high risk of Sarcopenia [15].

## Definition and cut off point

### Sarcopenia

We will use EWGSOP definition of sarcopenia to define the case group. According to this definition, individuals with abnormal ASM are considered as Pre-Sarcopenic. A Sarcopenic person is an individual who is identified as Pre-Sarcopenic and also has abnormal muscle strength or muscle performance. Those with all three abnormal criteria will be considered to be severely Sarcopenic [15].

### Metabolic syndrome

Metabolic syndrome will be defined as the presence of abdominal adiposity (wc> 94 cm in men and wc> 80 cm in women) plus any two of the following components as recommended by the International Diabetes Federation: low serum HDL cholesterol (< 40 mg/dl in men and < 50 in women) or specific treatment for this lipid abnormality, high serum triacylglycerol concentrations ≥ 150 mg/dl or lowering triglyceride drugs consumption, raised blood pressure ≥ 130/85 or treatment of previously diagnosed hypertension, elevated fasting plasma glucose ≥ 100 orpreviously diagnosed type 2 diabetes [32].

### Inflammatory marker

Studies have focused on the role of inflammatory cytokines especially IL6, TNFα, CRP in the progression of muscle loss in recent years [11,33] so we will measure this cytokine for both case and control group using appropriate kits.

### Dietary pattern

Unlike the traditional analyses which examine the relation between a single nutrient and a particular disease, dietary pattern analysis focuses on the relationship between diet and the risk of a disease. Dietary pattern is a better method to evaluate the association of nutrition and the risk of a disease for several reasons. First, each food is a combination of several nutrients. Second, the correlation among some nutrients is very high, so distinguishing the relationship between food and the disease will be difficult. Third, the effect of a single nutrient will be hard to detect due to the small amount, whereas the cumulative effect of nutrients in a dietary pattern may be large enough to detect [34]. All these have led to a growing interest among dietitians for using dietary pattern analysis to evaluate the relationship of food and chronic disease.

### Data processing & analysis

The nutritional intake of each food item will be converted to gram/day and dietary analysis will be conducted using the Iranian food composition table [35] and the USDA food composition data [36]. We will identify major dietary patterns using factor analysis and the FFQ data. To do so, we first group similar food items in the FFQ so that each food group is consumed by at least 10 participants. We will then use principle component analyses to calculate each group’s loading factor based on major dietary patterns. A summary score will be obtained for each pattern and will be used to investigate the relationship between dietary pattern and disease.

After examining the distributional assumption (normality) of data, we will compare the case and control groups using parametric tests as well as nonparametric methods. We will use the appropriate control methods and modeling techniques to eliminate the effect of confounding factors. The entire analysis will be done using SPSS software version 16 (SPSS Inc., Chicago IL) or STATA software.

## Conclusion

Sarcopeny is a multifactorial disease which is associated with a decrease in skeletal muscle mass and can be a major risk factor for development of frailty. It increases the elderly population’s disability and dependency and may impose high costs to health systems. Studying risk factors of Sarcopenia can be useful to prevent or delay the development of this disease.

There are plenty of studies in literature which state the risk factors associated with Sarcopenia in developed courtiers. However, not enough attention has been paid to this issue in developing countries. We will try to contribute to the literature by providing more information on the status of this disease and also its risk factors among elderly population in Iran. Due to the growing elderly population, attention to elderly health is one of the priorities of the health system in Iran. To our knowledge, this is the first study which focuses on Sarcopenia in community-dwelling elderly Iranians. We believe the results of our study can be useful to the public health system, Ministry of Health and Medical Education, and Seniors Health Administration. Our results can help policy makers prepare the necessary infrastructure for elderly health improvements and increase the quality of life in geriatric patients.

## Endnote

^a^According to 2006 Census, the district has a population of 237,292 which is about 3.3% of total population of Tehran.

## Competing interests

The authors declare that they have no competing interests.

## Authors’ contributions

RHa, conceived of the study and wrote draft of the manuscript. RHe, conceived of the study, participated in its design and revised the manuscript. AD, conceived of the study and revised the manuscript. MP, carried out the implementation and data collection of the pilot study and wrote the manuscript. AE, carried out consultation about the pilot study, FFQ preparation and data analyzing of pilot study. FB, carried out implementation and data collection of the pilot study. PP, carried out technical consultation about the laboratory assessment. FS, carried out consultation about the nutritional epidemiology. All authors read and approved the final manuscript.
